# Dietary Ferulic Acid Ameliorates Metabolism Syndrome-Associated Hyperuricemia in Rats *via* Regulating Uric Acid Synthesis, Glycolipid Metabolism, and Hepatic Injury

**DOI:** 10.3389/fnut.2022.946556

**Published:** 2022-06-30

**Authors:** Nanhai Zhang, Jingxuan Zhou, Lei Zhao, Ou Wang, Liebing Zhang, Feng Zhou

**Affiliations:** ^1^Beijing Key Laboratory of Functional Food From Plant Resources, College of Food Science and Nutritional Engineering, China Agricultural University, Beijing, China; ^2^Beijing Advanced Innovation Center for Food Nutrition and Human Health, Beijing Engineering and Technology Research Center of Food Additives, Beijing Technology and Business University, Beijing, China; ^3^National Institute for Nutrition and Health, Chinese Center for Disease Control and Prevention, Beijing, China

**Keywords:** ferulic acid, metabolism syndrome, hyperuricemia, uric acid synthesis, glycolipid metabolism, liver injury

## Abstract

Ferulic acid is a well-known phenolic acid compound and possesses multiple health-promoting and pharmacological effects. Metabolic syndrome (MetS) and hyperuricemia (HUA) have become health problems worldwide and are closely connected. The aim of this study was to explore the influence of ferulic acid on MetS-related HUA and its underlying mechanisms. Rats were administered high-fructose and high-fat diet (HFFD) with or without ferulic acid (0.05 and 0.1%) for 20 weeks. Intake of HFFD resulted in obesity, hyperglycemia, insulin resistance, and dyslipidemia, which were alleviated by ferulic acid consumption. Treatment of rats with ferulic acid diminished the levels of lipids and inflammatory cytokines and enhanced the activities of antioxidant enzymes in the liver caused by HFFD. Additionally, administration of ferulic acid blocked a HFFD-induced elevation in activities and mRNA expression of enzymes involving in uric acid (UA) synthesis. Molecular docking analysis denoted that ferulic acid bound to the active center of these enzymes, indicative of the potential interaction with each other. These two aspects might partially be responsible for the decrement in serum UA content after ferulic acid ingestion. In conclusion, ferulic acid supplementation ameliorated lipid and glucose metabolic abnormalities, hepatic damage, and UA formation in MetS rats. There was a dose correlation between lipid deposition and UA synthesis-related indicators. These findings implied that ferulic acid could be applied as a promising dietary remedy for the management of MetS-associated HUA.

## Introduction

Experimental studies have suggested that the long-term intake of high-fructose and high-fat diet (HFFD) that is the dietary pattern of modern people can result in metabolic syndrome (MetS), which has become a global health issue (1–3). MetS is a cluster of cardiometabolic abnormalities including hyperglycemia, hypertension, abdominal (visceral) obesity, hypertriglyceridemia, and low high-density lipoprotein-cholesterol (HDL-C) (4). Hyperuricemia (HUA), part of the cluster of metabolic disorders, is characterized by high serum uric acid (UA) level due to overproduction and/or less excretion of UA and then evokes gout and chronic kidney disease (5, 6). In the past few decades, the incidence of HUA has been successively rising worldwide. According to epidemiological surveys, the prevalence of HUA in Chinese adults remarkably rises from 8.4% in 2009–2010 to 14.0% in 2018–2019, and also more than 20.0% from 2007 to 2016 in United States (7, 8). Although the serum UA level is not a diagnostic criterion for MetS, longitudinal studies have demonstrated that HUA is closely associated with the development of MetS and its components, and there is a bidirectional relationship between them (9, 10). The morbidity of MetS gradually raises with the growth of serum UA level (11). Moreover, a cross-sectional study reported that about 32.9% of the very elderly population with HUA in Chengdu also developed MetS (12). The elevated serum UA level was also found in MetS animals and the UA content in HepG2 cells was significantly increased after treatment with HFFD (3, 13). Hence, HUA plays a critical role in the development of MetS and an inhibition of the serum UA level can not only prevent HUA, but also improve MetS to a certain extent (14, 15).

In recent years, UA-lowering strategies are focused on suppressing UA synthesis and promoting UA excretion (6). Reducing the production of UA using xanthine oxidase (XO) inhibitors is the first-line method for managing HUA (11). Allopurinol, febuxostat, and topiroxostat are the main XO inhibitors in clinical indication. They have been shown to be effective in decreasing the serum UA content and the risk of HUA and some cardiovascular events. However, their side effects are unignorable, such as gastrointestinal distress, allergic reaction, muscle pain, and impaired liver and renal function (16). Therefore, naturally occurring bioactive compounds have received considerable attention from researchers owing to their relatively low toxicity, and their prevention on MetS, type 2 diabetes mellitus, HUA, cardiovascular disease, cancer, and other diseases has been reported (17–19). Many literature reports have described that some bioactive substances, such as polyphenols and probiotics, could alleviate high- fructose-, high- fat-, or HFFD-induced glycolipid metabolic disorders, raised serum UA level, and hepatic injury (13, 20–22).

Ferulic acid is a well-known phenolic acid compound widely present in cereals, fruits, vegetables, and other edible plants (23). It is considered as an antioxidant and anti-inflammatory factor and as a result exerts numerous biological functions including modulating glucose tolerance and lipid metabolism, reducing the serum UA level, and ameliorating liver damage (24, 25). Wang et al. proved that ferulic acid supplementation (HFFD mixed with 0.05% ferulic acid) for 13 weeks could mitigate MetS in rats by alleviating HFFD-induced obesity, hyperglycemia, hyperlipidemia, insulin resistance (IR), inflammation, reduction of antioxidant activity, and impaired liver function (26). A previous study found that 90% wheat flour enriched with ferulic acid showed the same consequences in MetS rats fed with HFFD besides antioxidant activity and inflammation, and resulted in a decreasing tendency toward the serum UA level caused by HFFD (13). Recently, several studies exclusively explored the inhibitory mechanism of ferulic acid against XO *in vitro* by enzymatic and spectroscopic methods, which could help explain why the serum UA content declined caused by ferulic acid (27, 28). Hence, ferulic acid has a potential to improve MetS-HUA. In addition, the mechanism by which ferulic acid lowers the serum UA level in a MetS model caused by HFFD is poorly understood.

Overall, this work aimed to investigate the alleviating effect of ferulic acid on the MetS-related HUA in rats induced by HFFD and the potential mechanism of antihyperuricemic capacity. Based on previous animal experiments, the influence of a longer experimental period and a higher dose of ferulic acid on MetS-associated HUA was studied. Glucose tolerance, IR, lipid homeostasis, liver and kidney function, and oxidative stress, inflammation, UA synthesis in the liver were evaluated.

## Materials and Methods

### Chemicals

Ferulic acid (purity >98%) was obtained from Jingzhu Bio-Technology Co., Ltd. (Nanjing, China). Allopurinol was purchased from Shanghai Xinyi Wanxiang Pharmaceutical Co., Ltd. (Shanghai, China). Glucose was bought from Weifang Sheng Tai Pharmaceutical Co., Ltd. (Weifang, Shandong, China). Insulin injection was purchased from Novo Nordisk (China) Pharmaceuticals Co., Ltd. (Beijing, China). Commercial assay kits including glucose, total cholesterol (TC), triglycerides (TGs), HDL-C, low-density lipoprotein-cholesterol (LDL-C), free fatty acid (FFA), hemoglobin A1c (HbA1c), aspartate aminotransferase (AST), alanine aminotransferase (ALT), alkaline phosphatase (ALP), UA, blood urea nitrogen (BUN), and creatinine (CRE) were obtained from Biosino Bio-Technology and Science Inc. (Beijing, China). The assay kits for malondialdehyde (MDA), superoxide dismutase (SOD), catalase (CAT), glutathione peroxidase (GSH-Px), XO, adenylate kinase (AK), 5′-nucleotidase (5′-NT), adenosine deaminase (ADA), purine nucleoside phosphorylase (PNP), and lipopolysaccharide (LPS) and enzyme-linked immunosorbent assay (ELISA) kits for insulin, tumor necrosis factor-α (TNF-α), interleukin (IL)-1β, IL-18, and interferon-γ (IFN-γ) were obtained from Beijing Sinouk Institute of Biological Technology (Beijing, China). A BCA protein assay kit was supplied from Beyotime Biotechnology Inc. (Beijing, China). Other chemicals were of analytical grade.

### Experimental Animals

Male Sprague–Dawley rats (aged 8 weeks) were supplied by Vital River Laboratory Animal Technology Co., Ltd. (Beijing, China) [Certificate SCXK (Beijing) 2016-0006]. Rats were housed in separate cages under a 12–12 h light-dark cycle (8:00 a.m. to 8:00 p.m.) at an ambient temperature of 23 ± 2°C and relative humidity of 55 ± 5% and given free access to food and water. The animal experiment was approved by the Animal Ethics Committee of the Beijing Key Laboratory of Functional Food from Plant Resources. All animal procedures were performed in compliance with the guidelines for the care and use of laboratory animals of the National Institutes of Health.

After acclimated to the environment for 1 week, 50 rats were randomized into 5 groups (*n* = 10 per group) and fed with different diets for 20 weeks. These groups were as follows: (a) control group: basal diet (13.9 kJ/g, 4.5% fat, w/w); (b) model group: HFFD (18.9 kJ/g, 18% fructose and 20% lard); (c–d) ferulic acid group: HFFD mixed with ferulic acid; (e) allopurinol group (AP): HFFD mixed with allopurinol. The compositions of the basal diet and HFFD were the same as those in the previous study (Table 1) (13). Allopurinol was utilized as a positive control and added in the HFFD with a proportion of 0.0145% at its effective intragastric dosage (10 mg/kg body weight) from several reports (29–31). Ferulic acid was added in the HFFD with proportions of 0.05 and 0.1% (FAL and FAH), respectively. The dosage of ferulic acid was selected according to our preceding experiment (26). The body weight and food consumption were monitored weekly.

**TABLE 1:** Composition of basal diet and HFFD.

	Basal diet	HFFD
Corn starch (g/kg)	250	–
Wheat middling (g/kg)	200	–
Refined wheat flour (g/kg)	219	420
Soybean meal (g/kg)	200	80
Fructose (g/kg)	–	180
Lard (g/kg)	–	200
Chicken powder (g/kg)	–	80
Sodium cholate (g/kg)	–	2
Miscellaneous meal (g/kg)	45	–
Vegetable oil (g/kg)	20	–
Fish meal (g/kg)	20	–
Calcium bicarbonate (g/kg)	10	10
Mountain flour (g/kg)	16	8
Vitamin and mineral mixture (g/kg)	20	20
Total energy (kJ/g)	13.9	18.9

*HFFD, high-fat and high-fructose diet.*

### Oral Glucose and Insulin Tolerance Tests

Oral glucose tolerance test (OGTT) was performed using oral gavage of glucose (2 g/kg body weight) after 12 h of fasting on the first day of week 19, and insulin tolerance test (ITT) was conducted *via* intraperitoneal supplementation with insulin (0.5 U/kg body weight) after 4–5 h of fasting on the last day of week 19. The blood glucose concentrations were monitored by the tail blood using a glucometer (Life Scan Inc., Milpitas, CA, United States) with One Touch Ultra test trips at 0, 30, 60, and 90 min of gavage or injection. The integrated areas under the curve (AUC) of OGTT and ITT were calculated by a trapezoidal method (32).

### Sample Collection

At the end of the experimental period, the rats were fasted overnight with water *ad libitum* and sacrificed by cervical dislocation under deep anesthesia. Blood samples were collected by retro-orbital sinus puncture and incubated at 4°C for 6 h. Liver, kidney, perirenal fat, and epididymal fat tissues were dissected and weighed immediately for calculating the organ index [organ index (%) = organ weight/final body weight × 100%]. One part of liver tissues was soaked in a 10% formalin solution for histopathological observation. The remaining livers were flash-frozen in liquid nitrogen and stored at −80°C for further analysis.

### Detection of Biochemical Indicators

Serum samples were separated by centrifugation at 4000 × *g* for 20 min at 4°C. The serum lipids profiles (TG, HDL-C, LDL-C, and FFA), liver function markers (AST, ALT, and ALP), kidney function markers (UA, BUN, and CRE), and glucose metabolism markers [fasting blood glucose (FBG) and HbA1c] were determined on a Mindray BS-420 automatic biochemistry analyzer (Shenzhen Mindray Bio-Medical Electronics Co., Ltd., Shenzhen, China) using corresponding assay kits following the manufacturer’s instructions. The level of fasting blood insulin (FBI) at week 20 was measured using an ELISA kit. Homeostasis model assessment of insulin resistance (HOMA-IR), β-cell function (HOMA-β), and quantitative insulin sensitivity check index (QUICKI) were calculated as follows (33): HOMA-IR = FBG × FBI/22.5; HOMA-β = 20 × FBI/FBG-3.5; QUICKI = 1/[log (FBI) + log (FBG)].

The liver tissues were homogenized and the lipids were extracted according to the protocols of Zhang et al. (2). The concentrations of TG, TC, and FFA were detected using corresponding assay kits. The supernatant was collected from the liver homogenates after centrifugation at 12,000 × *g* for 20 min at 4°C for other hepatic parameters. The activities of SOD, CAT, GSH-Px, XO, AK, 5′-NT, ADA, and PNP, as well as the contents of MDA, UA, and LPS, were measured according to the instruction manuals of corresponding commercial kits. ELISA kits were used to determine the levels of TNF-α, IL-1β, IL-18, and IFN-γ in the liver homogenate. Total protein concentrations of the liver were measured by a BCA protein assay kit.

### RNA Isolation and Quantitative Real-Time PCR

Total RNA was extracted from the liver tissues using TRIpure reagent (Aidlab Biotechnologies Co., Ltd., Beijing, China) and reverse transcribed into cDNA was synthesized using a FastQuant RT kit (Tiangen Biotech Co. Ltd., Beijing, China). The mRNA expression levels were measured on a CFX96 real-time PCR detection system (CFX96, Bio-Rad, United States) using SuperReal PreMix Plus with SYBR Green (Tiangen Biotech Co. Ltd., Beijing, China). The primer sequences were synthesized by Sangon Biotech Co., Ltd. (Shanghai, China) and are provided in Table 2. The mRNA expression levels were normalized to GAPDH and calculated by the 2^–ΔΔ*Ct*^ method.

**TABLE 2:** The rat-specific primer sequences used for qRT-PCR.

Gene	Forward primer (5′–3′)	Reverse primer (5′–3′)
*Ak1*	CTCTTCCAACGGCTTCTT	CTCTTCTTGATGGTCTCCTC
*Nt5e*	GGCTATCTGAAGGTTGAGT	CCGAGTTCCTGTGTAGAATA
*Pnp*	ATCCGTGACCACATCAAC	GCCTTCTGCCTCATATCC
*Ada*	GTGGTGGCTATGGACTTG	GGCTTCATCCTCTATTGTGT
*Xdh*	GGATGAGGTTACTTGTGTTG	TTGTGATAATGGCTGGAAGA
*Gapdh*	GCTGCCTTCTCTTGTGAC	CTTGACTGTGCCGTTGAA

### Liver Histopathology

The liver tissues were fixed with 10% formalin solution, embedded in paraffin, sectioned, and stained with hematoxylin and eosin (H&E). The histopathological changes of liver sections were examined with a light microscope (BA-9000 L, Osaka, Japan).

### Molecular Docking

The molecular docking study was performed on the AutoDock v4.2 program to investigate the interaction between ferulic acid and UA synthesis-associated enzymes. The crystal structure of AK (PDB ID: 1z83), 5′-NT (PDB ID: 2XCW), PNP (PDB ID: 3K8O), ADA (PDB ID: 1VFL), and XO (PDB ID: 1FIQ) was obtained from the RCSB Protein Database Bank.^1^ The 3D structures of ferulic acid were acquired from PubChem database.^2^ Before docking, AutoDock tools were applied to pretreat the proteins *via* removing all of the water molecules, adding the hydrogen atoms, and computing the Gasteiger charges. The ligand (ferulic acid) was designated the rotatable bonds. In the docking pattern, the flexible ligand inserted into the rigid protein. A grid box with dimensions of 60 Å × 60 Å × 60 Å and a grid spacing of 0.375 Å was set up to embrace the active sites of corresponding proteins. Then the Lamarckian genetic algorithm with local search and number of runs (100 times) were defined to conduct docking calculations. The combination model with the lowest binding energy and the highest number of ligands was considered as the best docking result. PyMol 2.5.2 and LigPlot^+^ v.2.2 were used to depict the outputs.

### Statistical Analysis

All data are presented as mean ± standard deviation (SD). Prism 8.0 (GraphPad Software, San Diego, CA, United States) was applied to analysis the statistical significance between groups by unpaired *t*-test. A value of *p* < 0.05 was considered statistically significant.

## Results

### Effects of Ferulic Acid on Basic Parameters in HFFD-Fed Rats

**FIGURE 1:**
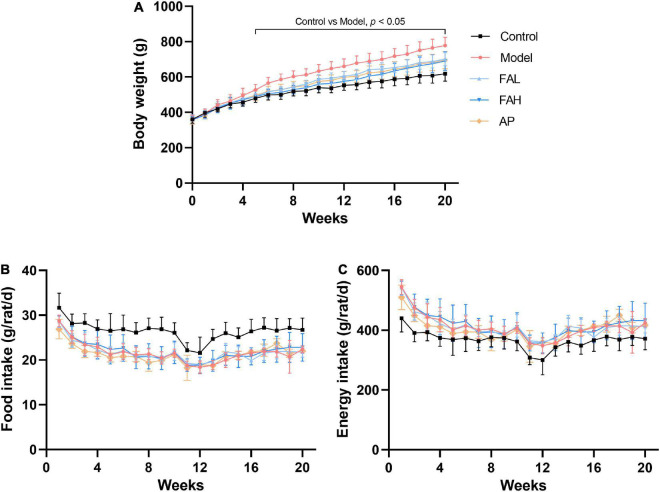
Effects ferulic acid of on changes of body weight **(A)**, food intake **(B)**, and energy intake **(C)** in HFFD-fed rats during experimental period. Data are presented as mean ± SD. A value of *p* < 0.05 was considered statistically significant. FAL, low dose of ferulic acid group administered of HFFD mixed with 0.05% ferulic acid; FAH, high dose of ferulic acid administered of HFFD mixed with 0.1% ferulic acid; AP, allopurinol group administered of HFFD mixed with 0.0145% allopurinol.

During the experimental period, the body weight of rats in different groups was increased gradually (Figure 1A). The food and energy intake declined slightly and then changed to be steady (Figures 1B,C). In the end, the final body weight and average energy intake in the treatment groups were raised and the average food intake was considerably reduced relative to those in the control group (*p* < 0.01, Table 3). Supplementation with low and high doses of ferulic acid and allopurinol markedly lowered the final body weight by 9.9, 11.3, and 10.6%, respectively (vs. Model, *p* < 0.05). However, the average food and energy intake was not affected by the treatment with ferulic acid and allopurinol.

**TABLE 3:** Effects of ferulic acid on body weight, food intake, and organ indexes in HFFD-fed rats.

Variable	Control	Model	FAL	FAH	AP
Initial body weight (g)	363.22 ± 21.84	367.46 ± 19.91	365.33 ± 23.46	365.20 ± 30.62	362.00 ± 26.00
Final body weight (g)	618.50 ± 42.14	777.78 ± 47.22**	700.83 ± 38.33**^,#^	690.00 ± 52.86**^,#^	695.71 ± 48.94**^,#^
Food intake (g/rat/day)	26.54 ± 2.25	21.85 ± 2.47**	21.90 ± 2.29**	22.23 ± 2.38**	21.53 ± 2.14**
Energy intake (g/rat/day)	368.88 ± 31.26	414.29 ± 46.87**	415.08 ± 43.35**	421.12 ± 45.00**	408.24 ± 40.55**
Liver index (%)	2.15 ± 0.12	2.38 ± 0.12**	2.14 ± 0.21^#^	2.06 ± 0.17^#^	2.21 ± 0.11^#^
Kidney index (%)	0.507 ± 0.024	0.424 ± 0.030**	0.453 ± 0.042**	0.488 ± 0.021^#^	0.461 ± 0.045*
Perirenal fat index (%)	0.48 ± 0.10	1.09 ± 0.10**	0.77 ± 0.16**^,#^	0.68 ± 0.13**^,#^	0.78 ± 0.10**^,#^
Epididymal fat index (%)	1.72 ± 0.23	2.91 ± 0.27**	2.29 ± 0.38**^,#^	2.21 ± 0.28**^,#^	2.53 ± 0.27**

Data are presented as mean ± SD. *p < 0.05, **p < 0.01, vs. Control; ^#^p < 0.05, vs. Model. FAL, low dose of ferulic acid group administered of HFFD mixed with 0.05% ferulic acid; FAH, high dose of ferulic acid administered of HFFD mixed with 0.1% ferulic acid; AP, allopurinol group administered of HFFD mixed with 0.0145% allopurinol.

HFFD caused an augment in the liver and fat indexes and a remarkable reduction in the kidney index of rats as comparison with the control group (*p* < 0.01, Table 3). Rats in the ferulic acid and AP groups produced the same results in the fat indexes (*p* < 0.01). After intake of ferulic acid (low and high doses) and allopurinol, these changes induced by HFFD were almost improved. The kidney index of rats was significantly enhanced only in the FAH group (vs. Model, *p* < 0.05). In addition to the FAH group, the kidney index of rats in the FAL and AP groups was lower than that in the control group (*p* < 0.05). No obvious variation was found in the liver index among the control, ferulic acid, and AP groups.

### Ferulic Acid Alleviated Glucose Metabolic Disorders in HFFD-Fed Rats

**FIGURE 2:**
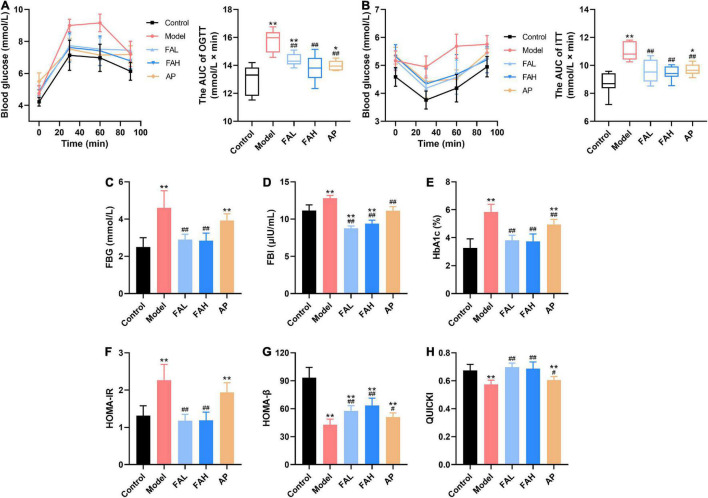
Effects of ferulic acid on glucose metabolism in HFFD-fed rats. **(A)** The AUC of OGTT; **(B)** the AUC of ITT; **(C)** FBG; **(D)** FBI; **(E)** HbA1c; **(F)** HOMA-IR; **(G)** HOMA-β; **(H)** QUICKI. Data are presented as mean ± SD. **p < 0.01, vs. Control; ^#^*p* < 0.05, ^##^*p* < 0.01, vs. Model. FAL, low dose of ferulic acid group administered of HFFD mixed with 0.05% ferulic acid; FAH, high dose of ferulic acid administered of HFFD mixed with 0.1% ferulic acid; AP, allopurinol group administered of HFFD mixed with 0.0145% allopurinol.

After HFFD administration for 20 weeks, the AUC of OGTT and ITT were markedly increased (vs. Control, *p* < 0.01, Figures 2A,B). The HOMA-IR index and levels of FBG, FBI, and HbA1c were remarkably raised and the values of HOMA-β and QUICKI were significantly diminished (vs. Control, *p* < 0.01, Figures 2C–H). These results indicated that the impaired glucose metabolism was found in the HFFD-fed rats. Allopurinol markedly decreased the AUC of OGTT and ITT (*p* < 0.01), concentrations of FBI and HbA1c (*p* < 0.01), and values of HOMA-β and QUICKI (*p* < 0.05). Only the FBI level in the AP group showed no apparent difference from that in the control group. All indices caused by HFFD were significantly ameliorated in the FAL and FAH groups (*p* < 0.01), which reached the level of the control group except HOMA-β value in both groups and the AUC of OGTT in the FAL group. Moreover, ferulic acid led to more minor FBI content than the control group (*p* < 0.01, Figure 2D).

### Ferulic Acid Suppressed Lipid Deposition in the Serum and Liver of HFFD-Fed Rats

**FIGURE 3:**
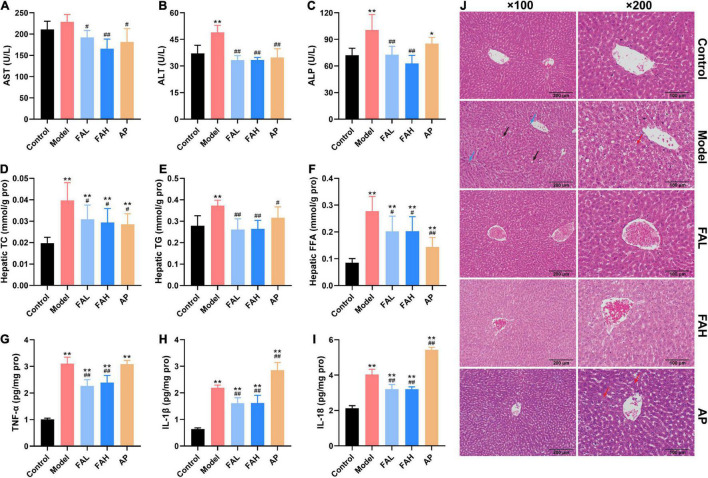
Effects of ferulic acid on hepatic function, lipid levels, inflammatory response, and histological changes in the liver of HFFD-fed rats. **(A)** Serum AST; **(B)** serum ALT; **(C)** serum ALP; **(D)** hepatic TC; **(E)** hepatic TG; **(F)** hepatic FFA; **(G)** hepatic TNF-α; **(H)** hepatic IL-1β; **(I)** hepatic IL-18; **(J)** representative H&E staining images in liver tissues (×100 and ×200 magnification). The blue, black, and red arrows represent inflammatory cells, fat vacuoles, and steatosis. Data are presented as mean ± SD. *p < 0.05, **p < 0.01, vs. Control; ^#^*p* < 0.05, ^##^*p* < 0.01, vs. Model. FAL, low dose of ferulic acid group administered of HFFD mixed with 0.05% ferulic acid; FAH, high dose of ferulic acid administered of HFFD mixed with 0.1% ferulic acid; AP, allopurinol group administered of HFFD mixed with 0.0145% allopurinol.

As shown in Figures 3D–F and Table 4, HFFD provoked a notable elevation in the contents of TC, TG, LDL-C, and FFA in the serum and liver and a diminishing in the serum HDL-C content relative to the control group (*p* < 0.05). The serum HDL-C and FFA levels in the FAL group and the TG level in the FAH and AP groups exhibited a similar trend as the model group, as well as the hepatic TC and FFA levels in the ferulic acid and AP groups. Compared with the model group, the concentrations of TC, TG, and FFA in the liver of rats treated with ferulic acid and allopurinol were markedly downregulated (*p* < 0.05, Figures 3D–F). Apart from the FFA content in the FAL group, administration of ferulic acid and allopurinol could alleviate the changes in the levels of TG, LDL-C, HDL-C, and FFA in the serum (*p* < 0.05, Table 4). Overall, the high dose of ferulic acid was superior to the low dose of ferulic acid and allopurinol in attenuating lipid accumulation in the serum and liver contributed by HFFD.

**TABLE 4:** Effects of ferulic acid on lipid profiles and renal indicators in the serum of HFFD-fed rats.

Variable	Control	Model	FAL	FAH	AP
**Lipid profiles**					
TG (mmol/L)	1.05 ± 0.23	2.30 ± 0.29**	1.39 ± 0.52^##^	1.41 ± 0.18*^,#^	1.57 ± 0.33*^,##^
LDL-C (mmol/L)	0.58 ± 0.10	0.77 ± 0.18*	0.51 ± 0.03^#^	0.50 ± 0.04^#^	0.42 ± 0.05^##^
HDL-C (mmol/L)	1.10 ± 0.12	0.78 ± 0.06**	0.94 ± 0.13*^,#^	0.99 ± 0.07^##^	0.94 ± 0.17^#^
FFA (mmol/L)	0.75 ± 0.09	1.08 ± 0.12**	0.94 ± 0.20*	0.75 ± 0.08^##^	0.80 ± 0.10^##^
**Renal indicators**					
UA (μmol/L)	62.46 ± 2.31	91.27 ± 11.90**	75.82 ± 9.74**^,#^	65.81 ± 7.22^##^	75.54 ± 3.07**^,#^
BUN (mg/L)	93.66 ± 7.81	160.01 ± 10.75**	91.31 ± 10.21^##^	100.82 ± 7.64^##^	79.51 ± 7.92*^,##^
CRE (μmol/L)	55.65 ± 1.77	57.09 ± 3.00	55.54 ± 2.62	54.83 ± 2.75	51.04 ± 1.93^##^

Data are presented as mean ± SD. *p < 0.05, **p < 0.01, vs. Control; ^#^p < 0.05, ^##^p < 0.01, vs. Model. FAL, low dose of ferulic acid group administered of HFFD mixed with 0.05% ferulic acid; FAH, high dose of ferulic acid administered of HFFD mixed with 0.1% ferulic acid; AP, allopurinol group administered of HFFD mixed with 0.0145% allopurinol.

### Ferulic Acid Improved Liver and Kidney Function in HFFD-Fed Rats

The activities of AST, ALT, and ALP and the contents of UA, BUN, and CRE in the serum were determined to evaluate the effects of ferulic acid on liver and kidney function in HFFD-fed rats (Figures 3A–C and Table 4). After consuming HFFD for 20 weeks, hepatic and renal injury occurred in rats according to the higher activities of ALT and ALP and levels of UA and BUN than rats in the control group (*p* < 0.01, Figures 3B,C and Table 4). But the AST activity and CRE content were not significantly affected by HFFD. The UA content in the FAL and AP groups and the ALP activity in the AP group were remarkably raised compared to those in the control group (*p* < 0.05). There were no obvious differences in other indices among the control, ferulic acid, and AP groups. In contrast to the model group, all indicators except the CRE content made an apparent improvement in the ferulic acid and AP groups (*p* < 0.05). The UA content in the FAL, FAH, and AP groups was dramatically decreased by 16.9, 27.9, and 17.2%, respectively (vs. Model, *p* < 0.05, Table 4). In addition, the high dose of ferulic acid exerted more efficient effects on reversing a HFFD-induced reduction of kidney function for the low dose of ferulic acid and allopurinol.

### Ferulic Acid Attenuated Inflammatory Response and Histological Changes in the Liver of HFFD-Fed Rats

The alterations of proinflammatory cytokines (TNF-α, IL-1β, and IL-18) in rat livers were presented in Figures 3G–I to assess the effects of ferulic acid on hepatic inflammatory response in HFFD-fed rats. Compared with the control group, an apparent increment of the contents of TNF-α, IL-1β, and IL-18 was observed in the model group by 3.1-, 3.4-, and 1.9-fold (*p* < 0.01). Although ferulic acid consumption also significantly enhanced these indicators compared to the control group (*p* < 0.01), the levels of TNF-α, IL-1β, and IL-18 showed a marked decrement after supplementation with ferulic acid (vs. Model, *p* < 0.01). Conversely, the IL-1β and IL-18 contents in the AP group were higher than those in the model group (*p* < 0.01). The results revealed that ferulic acid exhibited an alleviating effect in HFFD-caused inflammation in rat livers, while allopurinol could aggravate the hepatic inflammatory response.

Hematoxylin and eosin staining was used to analyze the effect of ferulic acid on the hepatic histopathological changes resulting from HFFD (Figure 3J). Rat livers in the control group had the expected regular architecture with central vein. Nevertheless, HFFD feeding for 20 weeks elicited severe damages in liver structure, characterized by disordered hepatocellular arrangement, inflammatory cell infiltration, and lots of fat vacuoles. The hepatic degeneration was normalized by ferulic acid administration relative to the model group as the infiltration of inflammatory cells and fat vacuoles were greatly alleviated. On the contrary, diffuse steatosis was also observed in the AP group.

### Ferulic Acid Mitigated Oxidative Stress in the Liver of HFFD-Fed Rats

**FIGURE 4:**
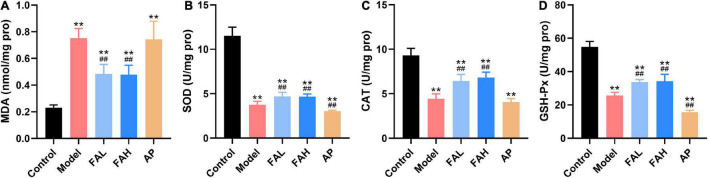
Ferulic acid suppressed lipid peroxidation and oxidative stress in the liver of HFFD-fed rats. **(A)** MDA; **(B)** SOD; **(C)** CAT; **(D)** GSH-Px. Data are presented as mean ± SD. **p < 0.01, vs. Control; ^##^*p* < 0.01, vs. Model. FAL, low dose of ferulic acid group administered of HFFD mixed with 0.05% ferulic acid; FAH, high dose of ferulic acid administered of HFFD mixed with 0.1% ferulic acid; AP, allopurinol group administered of HFFD mixed with 0.0145% allopurinol.

As displayed in Figure 4, rats administered HFFD with or without ferulic acid and allopurinol produced higher MDA level and lower activities of SOD, CAT, and GSH-Px for the rats in the control group (*p* < 0.01). Surprisingly, less SOD and GSH-Px activities were also found in the AP group than those in the model group (*p* < 0.01), along with no obvious changes in the MDA level and CAT activity as comparison with the model group. Treatment with ferulic acid conspicuously suppressed the MDA content and promoted the activities of antioxidant enzymes (vs. Model, *p* < 0.01).

### Ferulic Acid Inhibited Uric Acid Synthesis in the Liver of HFFD-Fed Rats

**FIGURE 5:**
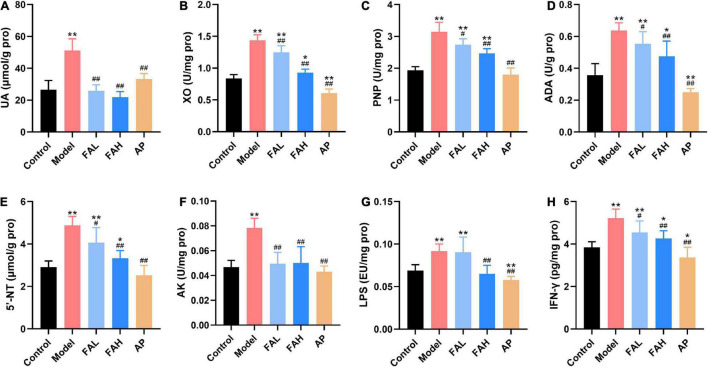
Effects of ferulic acid on the UA level and UA synthesis-associated indices in the liver of HFFD-fed rats. **(A)** UA; **(B)** XO; **(C)** PNP; **(D)** ADA; **(E)** 5′-NT; **(F)** AK; **(G)** LPS; **(H)** IFN-γ. Data are presented as mean ± SD. *p < 0.05, **p < 0.01, vs. Control; ^#^*p* < 0.05, ^##^*p* < 0.01, vs. Model. FAL, low dose of ferulic acid group administered of HFFD mixed with 0.05% ferulic acid; FAH, high dose of ferulic acid administered of HFFD mixed with 0.1% ferulic acid; AP, allopurinol group administered of HFFD mixed with 0.0145% allopurinol.

The contents of UA, IFN-γ, and LPS and activities of XO, PNP, ADA, 5′-NT, and AK in the liver were determined to investigate the effect of ferulic acid on hepatic UA synthesis of HFFD-fed rats (Figure 5). These indices in the model group were markedly increased with respect to those in the control group (*p* < 0.01). In the conditions of the high dose of ferulic acid and allopurinol, the rats provoked less UA level and UA synthesis-related indices in the liver compared to the rats receiving HFFD (*p* < 0.01). Rats in the FAL group showed the same results except for the LPS level. Additionally, the UA content and activities of PNP, 5′-NT, and AK in the AP group exhibited no significant changes from those in the control group, and allopurinol markedly lowered other indicators (vs. Control, *p* < 0.05). The AK activity and levels of UA and LPS in the FAH group reached the level of the control group, as well as the UA level and AK activity in the FAL group. Therefore, the high dose of ferulic acid possessed more beneficial effects on suppressing the UA synthesis-related indicators than the low dose of ferulic acid.

**FIGURE 6:**
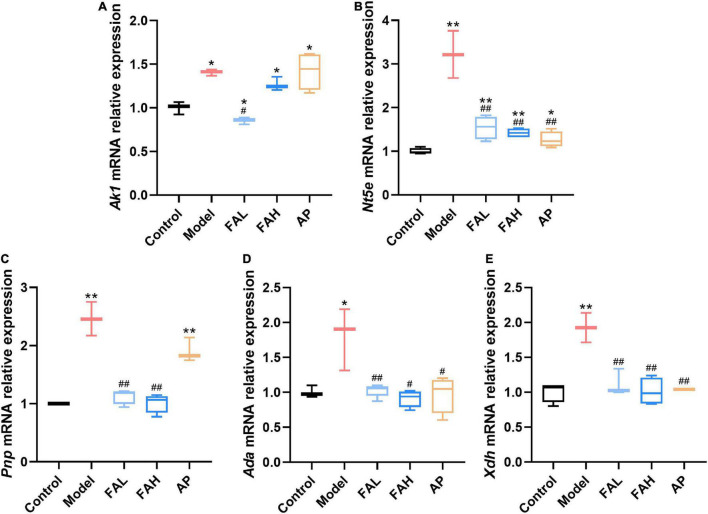
Effects of ferulic acid on the mRNA expression levels of UA synthesis-associated enzymes in the liver of HFFD-fed rats. **(A)** *Ak1*; **(B)** *Nt5e*; **(C)** *Pnp*; **(D)** *Ada*; **(E)** *Xdh*. Data are presented as mean ± SD. *p < 0.05, **p < 0.01, vs. Control; ^#^*p* < 0.05, ^##^*p* < 0.01, vs. Model. FAL, low dose of ferulic acid group administered of HFFD mixed with 0.05% ferulic acid; FAH, high dose of ferulic acid administered of HFFD mixed with 0.1% ferulic acid; AP, allopurinol group administered of HFFD mixed with 0.0145% allopurinol.

To further explore the underlying inhibitory mechanisms of ferulic acid on the activities of UA synthesis-associated enzymes, the mRNA expression levels of these enzymes in rat livers were determined (Figure 6). Intake of HFFD upregulated the relative expression levels of *Ak1*, *Nt5e*, *Pnp*, *Ada*, and *Xdh* genes (*p* < 0.05), and ferulic acid and allopurinol could counteract this augment. No obvious difference in the relative expressions of *Pnp*, *Ada*, and *Xdh* mRNA was noted between the ferulic acid and control groups, and the *Ak1* gene expression level was markedly lower in the FAL group (vs. Control, *p* < 0.05). Administration of the low dose of ferulic acid considerably reduced the mRNA expression of all genes as compared with the HFFD group (*p* < 0.05). Apart from the *Ak1* gene expression level, the mRNA expression of other genes also declined after supplementation with the high dose of ferulic acid compared to that in the model group (*p* < 0.05). Except for the relative expressions of *Ak1* and *Pnp* mRNA, allopurinol resulted in a remarkable downregulation in the relative expression of other genes (vs. Model, *p* < 0.05).

### Molecular Docking Analysis

**FIGURE 7:**
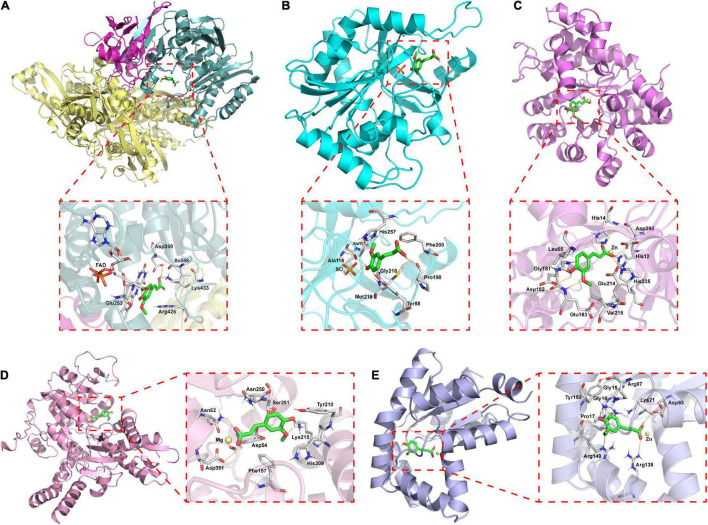
Docking model of the best binding pose for ferulic acid with UA synthesis-associated enzymes and three-dimensional structure diagrams for the interaction of ferulic acid with the major amino acid residues of the enzymes. **(A)** XO; **(B)** PNP; **(C)** ADA; **(D)** 5′-NT; **(E)** AK.

**FIGURE 8:**
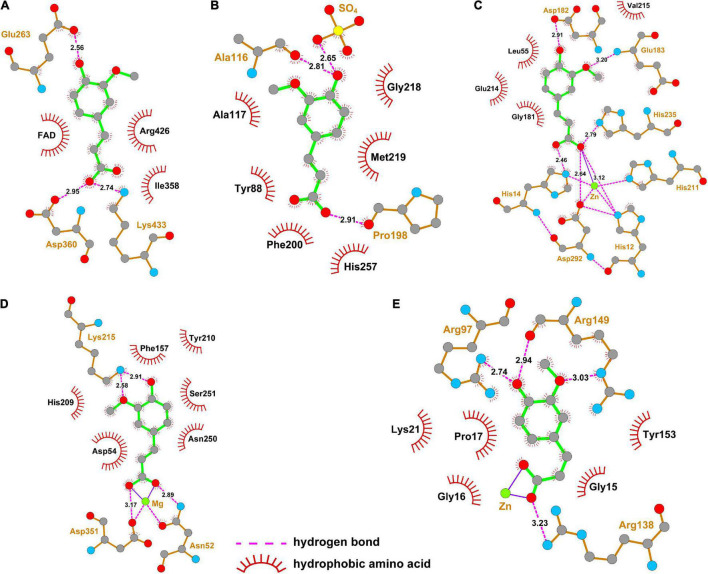
Two-dimensional structure diagrams for the interaction of ferulic acid with the major amino acid residues of UA synthesis-associated enzymes. **(A)** XO; **(B)** PNP; **(C)** ADA; **(D)** 5′-NT; **(E)** AK.

The interaction of ferulic acid with UA synthesis-associated enzymes could be another factor that ferulic acid suppressed the activities of UA synthesis-associated enzymes. Molecular docking is widely used to visualize the interaction and predict the possible binding models of ligand-protein complexes. According to the docking study, the estimated free energies of ferulic acid binding with XO, PNP, ADA, 5′-NT, and AK were −23.73, −16.74, −38.01, −50.98, and −52.87 kJ/mol, respectively. As displayed in Figure 7A, ferulic acid bound to the active cavity at the center of the flavin adenine dinucleotide (FAD) region of XO. Figure 8A demonstrated that ferulic acid formed three hydrogen bonds with the residues Glu263, Asp360, and Lys433 (2.56, 2.95, and 2.74 Å, respectively), and interacted with the residues Ile358 and Arg426 through hydrophobic forces. Figures 7B, 8B showed that ferulic acid inserted into the active pocket of PNP by forming hydrogen bonds between the carboxyl and phenolic hydroxyl groups of ferulic acid and PNP with Pro198, Ala116 residues and -SO_4_ (2.91, 2.81, and 2.65 Å, respectively). The stability of the ferulic acid-PNP complex was also strengthened by hydrophobic interactions with the amino acid residues Tyr88, Ala117, Phe200, Gly218, Met219, and His257.

Figures 7C–E revealed that ferulic acid was docked into the active sites of ADA, 5′-NT, and AK. Ferulic acid formed six hydrogen bonds with the residues His12, His14, Asp182, Glu183, His211, His235, and Asp292 of ADA (Figure 8C). Some residues, including Leu55, Gly181, Glu214, and Val215, were also involved in stabilizing the conformation of the ferulic acid-ADA complex through hydrophobic forces (Figure 8C). Moreover, a key coordination bond was observed between one of the oxygens of the carboxyl group of ferulic acid and catalytic zinc ion of the ADA active site which in turn interacted with His12, His211, and Asp292 residues (Figure 8C). The negatively charged carboxyl group of ferulic acid could also produce a coulombic interaction with the positively charged zinc ion (Figure 8C). The ferulic acid-5′-NT complex was stabilized by three hydrogen bonds with the residues Asn52, Lys215, and Asp351 and hydrophobic effects with the residues Asp54, Phe157, His209, Tyr210, Asn250, and Ser251 (Figure 8D). Ferulic acid made three hydrogen bonds with Arg97, Arg138, and Arg149 residues of AK and hydrophobic interactions with Gly15, Gly16, Pro17, Lys21, and Tyr153 residues (Figure 8E). There was a coordination bond between the carboxyl group of ferulic acid with magnesium ion and zinc ion which were the active site of 5′-NT and AK, respectively (Figures 8D,E).

## Discussion

After prolonged ingestion of HFFD, an increasing number of people suffer from MetS, diabetes mellitus, cardiovascular disease, and other chronic diseases, which are closely linked to HUA (9, 32). Under the circumstances of most drugs that show some side effects, dietary intervention derived from natural ingredients exhibits a great advantage (16, 17, 34). Our team successfully established a MetS model using HFFD *in vivo* and reported that several polyphenols and polyunsaturated fatty acids could alleviate MetS (2, 32, 35). Ferulic acid, one of the phenolic acid compounds, possessed a beneficial function in regulating glucose and lipid metabolism, antioxidation, antiinflammation, etc. (24, 26). The UA level and activity of XO, a crucial rate-limiting enzyme in the UA synthesis process, can be suppressed by ferulic acid, making ferulic acid show a potential antihyperuricemic property (13, 27). Whereas, little information is currently available on the underlying mechanism of ferulic acid on the UA content in a MetS model. Thus, this study set out with the aim of evaluating the protective effect of ferulic acid on MetS-related HUA in rats fed with HFFD for 20 weeks, as well as its underlying mechanism.

A Joint Scientific statement points out that people with three or more abnormal manifestations can be diagnosed with MetS (4). Abdominal obesity is an uppermost feature among five components of MetS and characterized by excessive visceral fat accumulation (36). In the present study, compared with the control group, long-term HFFD consumption significantly enhanced the energy intake and fat indexes, and thus accelerated the final body weight (Table 3). Although supplementation with ferulic acid and allopurinol had no effect on the energy intake, fat deposition and the final body weight were markedly diminished in the ferulic acid and AP groups (vs. Model). The high dose of ferulic acid (HFFD with 0.1% of ferulic acid) showed even more efficiency in lowering abdominal obesity (perirenal fat index decreased 37.6% and epididymal fat index decreased 24.1%) than allopurinol and the low dose of ferulic acid. It has been reported that ferulic acid could reduce adipogenesis, stored lipid content in adipose tissue and adipose tissue expansion, and stimulate white-fat browning *via* regulating the expression of related genes, revealing the anti-obesity effect of ferulic acid (37–39). We found that allopurinol, a XO inhibitor, also exerted anti-obesity capacity, which was in line with the studies of Cho et al. (40) and Zhang et al. (41).

When adipose tissue is dysfunctional and hypertrophic, the proinflammatory cytokines are secreted from adipose tissue, further contributing to impaired glucose metabolism like hyperglycemia and IR which are important features of MetS (36, 39, 42). FBG, HbA1c, and OGTT were used to assess glucose homeostasis, and FBI, HOMA-IR, HOMA-β, and QUICKI were used to evaluate insulin sensitivity (33). Our work showed that HFFD led to glucose metabolic disorders in rats, on the basis of the determination of FBG, HbA1c, OGTT, ITT, FBI, HOMA-IR, HOMA-β, and QUICKI (Figure 2). Whereas, treatment with allopurinol could only increase glucose tolerance and insulin sensitivity to control the long-term glycemic concentration (HbA1c) (43) and make the FBG level a decreasing trend (vs. Model). Ferulic acid intake effectively maintained glucose homeostasis close to normal level by improving glucose tolerance and insulin sensitivity in peripheral tissues and mitigating IR in the liver. There was a dose correlation in glucose tolerance between low and high doses of ferulic acid. There is evidence to suggest that ferulic acid blunted HFFD-induced glucose production probably by an inactivation of forkhead box 1 in livers and a suppression of downstream enzymes, phosphoenolpyruvate carboxylase, and glucose-6-phosphatase, thus inhibiting the hepatic gluconeogenesis (38). In addition, administration of ferulic acid could cause a downregulation of glucose transporter 2 gene expression to weaken glucose output, ultimately reducing the FBG content (44).

The majority of studies have reported that visceral obesity and IR are recognized to have a causal relationship with the development of dyslipidemia, which is characterized by higher serum TC, TG, FFA, and LDL-C levels, and the lower HDL-C level (45–47). The current results elucidated that abnormal lipid metabolism occurred in the model group, but was attenuated to a varying degree after supplementation with ferulic acid and allopurinol (Table 4), exhibiting the hypolipidemic effect of ferulic acid and allopurinol. These findings are consistent with our previous study (26) and the literature of Mostafa-Hedeab et al. (48), which was likely due to the role of ferulic acid and allopurinol in improving obesity and IR. The liver plays a crucial role in glucose and lipid metabolism (49). Multiple studies confirm a positive association between excess dietary carbohydrates and fats and the risk of non-alcoholic fatty liver disease, which can produce some characteristics observed in individuals with MetS (50, 51). The circulatory lipids like FFA from adipocytes can be taken up by the hepatocytes for the synthesis of TG and other lipids, and fructose metabolism can also lead to lipid deposition in the live, bringing about non-alcoholic fatty liver and then progress to non-alcoholic steatohepatitis, fibrosis, cirrhosis, hepatocellular cancer, and liver failure (49, 52, 53). In the present study, we discovered that rats treated with ferulic acid and allopurinol showed less hepatic steatosis than rats in the model group, embodied in lower hepatic TG, TC, and FFA contents (Figures 3D–F), likely though suppressing *de novo* lipogenesis and promoting β-oxidation of FFA (26, 39, 40).

Excessive lipid accumulation in the liver could aggravate the lipid peroxidation and generation of reactive oxygen species, subsequently triggering inflammatory pathways and in turn decreasing insulin sensitivity (54). Consequently, treatment of rats with ferulic acid obviously decreased the production of MDA (a lipid peroxidation marker) and enhanced the activities of antioxidant enzymes (SOD, CAT, and GSH-Px) (Figure 4) which could scavenge free radicals (55), contributing to a mitigation of lipid peroxidation and oxidative stress in the liver caused by HFFD. Additionally, the levels of TNF-α, IL-1β, and IL-18 in rat livers were significantly reduced after ferulic acid consumption without a dose relationship (Figures 3G–I), which coincided with the result of IR. These results suggested that ferulic acid alleviated the hepatic inflammation resulting from HFFD because of the diminished oxidative stress. However, lower activities of antioxidant enzymes and higher contents of inflammatory cytokines were observed in the AP group than those in the model group (Figures 3H,I, 4B,D), indicating that a long-term diet with allopurinol could exacerbate HFFD-induced hepatic oxidative stress and inflammation and had an adverse effect on the liver. H&E staining analysis in rat lives produced the same consequence that fat vacuoles and inflammatory cells were lessened by ferulic acid treatment (Figure 3J).

The liver is also the main place of UA production through fructose metabolism. The majority of studies have found that high fructose ingestion has a substantial relationship with the risk of hyperuricemia and gout (56). Dietary fructose is predominantly absorbed from the intestine *via* hexose transporter SLC2A5 and then extracted from peripheral plasma to the liver in large part *via* glucose transporter SLC2A2 for metabolism (57). In the liver tissues, fructose is phosphorylated to fructose-1-phosphate by fructokinase (KHK), accompanied by the reduction of ATP and intracellular phosphate. ADP is produced and then transformed into AMP by AK. The rapid depletion of phosphate can activate AMP deaminase for converting AMP to inosine monophosphate. AMP and inosine monophosphate are metabolized to hypoxanthine in the response of 5′-NT, ADA, and PNP. UA is ultimately generated under the effect of XO (56). The production of UA may provoke hepatic lipid accumulation by stimulating the mitochondrial oxidative stress and potentiating the lipogenic effects of fructose through upregulating the KHK expression, thus causing IR and inflammation (58). Excessive UA may also result in the development and progression of renal disease (59). Therefore, inhibiting the activities of UA synthesis-related enzymes to reduce the serum UA level is conducive to diminishing the risk of HUA, MetS, and chronic kidney disease. In the current study, supplementation with ferulic acid and allopurinol markedly alleviated HFFD-induced HUA through weakening the UA content in the serum and liver (Table 4 and Figure 5A), further ameliorating hepatic lipid and glucose metabolic disorders. In particular, the high dose of ferulic acid (HFFD with 0.1% of ferulic acid) shows a more beneficial effect in UA lowering than the low dose of ferulic acid and allopurinol. Concomitant with the UA content, treatment with ferulic acid and allopurinol ameliorated renal injury caused by HFFD according to the lower BUN concentration in the serum than that in the model group (Table 4).

Accumulating evidence has suggested that natural compounds could downregulate the XO and ADA activities to mitigate the UA formation, such as astaxanthin, konjac glucomannan, and black tea with fungal growth (60–62). However, the changes in the activities of other UA synthesis-related enzymes induced by natural substances are poorly investigated. The current study found that the activities of AK, 5′-NT, ADA, PNP, and XO in the rat lives were elevated by HFFD intake (vs. Control), while the growth of their activities was blocked by allopurinol consumption (Figures 5B–F), resulting in the decrease of the UA level. Rats after consuming ferulic acid produced the same and less efficient effects as allopurinol with a dose correlation. These data manifested that ferulic acid and allopurinol blunted the UA synthesis *via* suppressing the activities of related enzymes. The decrement of enzyme activity is probably because of the interaction of the enzyme with other molecules and the downregulation of the enzyme expression. An increasing number of *in vitro* studies have reported that polyphenols could bind to ADA, PNP, and XO to inhibit their activities (63–66). Polyphenols, dietary fibers, and probiotics have been proved to reduce the mRNA expression of *Ada* and *Xdh* in HUA animals (60, 61, 67). Similarly, molecular docking analysis in this work exhibited that ferulic acid inserted into the corresponding active center of AK, 5′-NT, ADA, PNP, and XO (Figure 7), having a potential to interact with these enzymes. Intake of HFFD upregulated the gene expressions of AK (coded by *Ak1*), 5′-NT (coded by *Nt5e*), PNP (coded by *Pnp*), ADA (coded by *Ada*), and XO (coded by *Xdh*), whereas fewer mRNA expressions of these genes in the ferulic acid and AP groups were observed with a dose correlation compared to the model group (Figure 6). These consequences suggested that the potential interaction and lowering the mRNA expression may be the reason for the diminished activities of AK, 5′-NT, ADA, PNP, and XO in the presence of ferulic acid. Moreover, the contents of LPS, IL-1β, and IFN-γ in rat livers, which were significantly raised after HFFD intake, were decreased by ferulic acid and allopurinol treatment (Figures 3H, 5G,H). Scientific evidence has demonstrated that the XO mRNA level was enhanced by LPS, IL-1β, and IFN-γ in hepatocytes (68, 69). The release of bacterial LPS is derived from intestinal barrier dysfunction and triggers hepatic inflammatory response through stimulating the proinflammatory factors including IL-1β and IFN-γ (67, 70). As a result, the normalization of XO mRNA expression after ferulic acid ingestion may partially be caused by endotoxin LPS and downstream IL-1β and IFN-γ. The consequence is in agreement with the report of Wang et al. that *Lactobacillus brevis* DM9218 reverted a fructose-induced elevation of XO expression *via* blunting the release of LPS and levels of IL-1β and IFN-γ (20).

**FIGURE 9:**
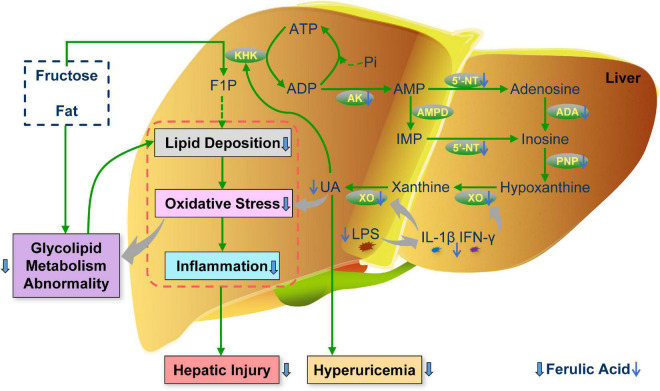
Schematic illustration of the proposed regulatory mechanism of ferulic acid in MetS-related HUA. F1P, fructose-1-phosphate; IMP, inosine monophosphate; UA, uric acid; KHK, fructokinase; AK, adenylate kinase; AMPD, AMP deaminase; 5′-NT, 5′-nucleotidase; ADA, adenosine deaminase; PNP, purine nucleoside phosphorylase; LPS, lipopolysaccharide; IL-1β, interleukin-1β; IFN-γ, interferon-γ.

Overall, this study illuminated that ferulic acid ingestion for 20 weeks counteracted aberrant lipid and glucose metabolism and liver injury (including lipid deposition, oxidative stress, and inflammation) in HFFD-fed rats, thus attenuating MetS. In addition, ferulic acid conferred improving effects against MetS-related HUA *via* inhibiting the production of UA. The decrement of the UA level could be attributed to the potential interaction between ferulic acid and UA synthesis-associated enzymes by molecular docking, and the lowering gene expression levels of these enzymes. An illustrated overview of the proposed regulatory mechanism of ferulic acid in MetS-related HUA is displayed in Figure 9. Furthermore, the high dose of ferulic acid (HFFD mixed with 0.1% ferulic acid) possessed more efficient functions in lipid accumulation and UA synthesis-related indices than the low dose of ferulic acid (HFFD mixed with 0.05% ferulic acid). Herein, our findings divulged that ferulic acid might become an ideal nutritious supplementary against MetS-associated HUA. The precise interaction between ferulic acid and UA synthesis-associated enzymes and mechanisms for modulating the UA excretion and gut microbiota were investigated in the future.

## Data Availability Statement

The original contributions presented in this study are included in the article/supplementary material, further inquiries can be directed to the corresponding author.

## Ethics Statement

The animal study was reviewed and approved by Ethics Committee of the Beijing Key Laboratory of Functional Food from Plant Resources.

## Author Contributions

NZ: investigation, project administration, formal analysis, visualization, and writing – original draft. JZ: project administration. LeZ: supervision. OW and LiZ: resources. FZ: conceptualization, supervision, funding acquisition, and writing – review and editing. All authors contributed to the article and approved the submitted version.

## Conflict of Interest

The authors declare that the research was conducted in the absence of any commercial or financial relationships that could be construed as a potential conflict of interest.

## Publisher’s Note

All claims expressed in this article are solely those of the authors and do not necessarily represent those of their affiliated organizations, or those of the publisher, the editors and the reviewers. Any product that may be evaluated in this article, or claim that may be made by its manufacturer, is not guaranteed or endorsed by the publisher.

## Footnotes

1
http://www.rcsb.org/pdb

2
https://pubchem.ncbi.nlm.nih.gov/
